# Primary renal sarcomas: imaging features and discrimination from non-sarcoma renal tumors

**DOI:** 10.1007/s00330-021-08201-4

**Published:** 2021-07-31

**Authors:** Johannes Uhlig, Annemarie Uhlig, Sophie Bachanek, Mehmet Ruhi Onur, Sonja Kinner, Dominik Geisel, Michael Köhler, Heike Preibsch, Michael Puesken, Dominik Schramm, Matthias May, Pieter De Visschere, Marc-André Weber, Alexey Surov

**Affiliations:** 1 Department of Diagnostic and Interventional Radiology, University Medical Center Goettingen, Robert-Koch-Strasse 40, 37075 Goettingen, Germany; 2 Section of Interventional Radiology, Yale School of Medicine, New Haven, CT USA; 3 Department of Urology, University Medical Center Goettingen, Goettingen, Germany; 4 Institute of Urologic Oncology, University of California at Los Angeles, Los Angeles, CA USA; 5 Department of Radiology, University of Hacettepe School of Medicine, Ankara, Turkey; 6 Institute for Diagnostic and Interventional Radiology, University of Essen, Essen, Germany; 7 Department of Radiology, Charité, Berlin, Germany; 8 Department of Radiology, University of Muenster, Muenster, Germany; 9 Department of Radiology, University of Tuebingen, Tuebingen, Germany; 10 Department of Diagnostic and Interventional Radiology, University Hospital Cologne, Cologne, Germany; 11 Department of Radiology, Martin-Luther-University Halle-Wittenberg, Halle, Germany; 12 Department of Radiology, University Hospital Erlangen, Erlangen, Germany; 13 Department of Radiology and Nuclear Medicine, Division of Genitourinary Radiology and Mammography, Ghent University Hospital, Ghent, Belgium; 14 Institute of Diagnostic and Interventional Radiology, Pediatric Radiology and Neuroradiology University Medical Center of Rostock, Rostock, Germany; 15 Department of Diagnostic and Interventional Radiology, University Hospital of Heidelberg, Heidelberg, Germany; 16 Department of Radiology and Nuclear Medicine, Otto-von-Guericke University, Magdeburg, Germany; 17 Department of Radiology, University of Leipzig, Leipzig, Germany

**Keywords:** Renal cancer, Renal sarcoma, Radiological imaging, Machine learning

## Abstract

**Objectives:**

To assess imaging features of primary renal sarcomas in order to better discriminate them from non-sarcoma renal tumors.

**Methods:**

Adult patients diagnosed with renal sarcomas from 1995 to 2018 were included from 11 European tertiary referral centers (Germany, Belgium, Turkey). Renal sarcomas were 1:4 compared to patients with non-sarcoma renal tumors. CT/MRI findings were assessed using 21 predefined imaging features. A random forest model was trained to predict “renal sarcoma vs. non-sarcoma renal tumors” based on demographics and imaging features.

**Results:**

*n* = 34 renal sarcomas were included and compared to *n* = 136 non-sarcoma renal tumors. Renal sarcomas manifested in younger patients (median 55 vs. 67 years, *p* < 0.01) and were more complex (high RENAL score complexity 79.4% vs. 25.7%, *p* < 0.01). Renal sarcomas were larger (median diameter 108 vs. 43 mm, *p* < 0.01) with irregular shape and ill-defined margins, and more frequently demonstrated invasion of the renal vein or inferior vena cava, tumor necrosis, direct invasion of adjacent organs, and contact to renal artery or vein, compared to non-sarcoma renal tumors (*p* < 0.05, each). The random forest algorithm yielded a median AUC = 93.8% to predict renal sarcoma histology, with sensitivity, specificity, and positive predictive value of 90.4%, 76.5%, and 93.9%, respectively. Tumor diameter and RENAL score were the most relevant imaging features for renal sarcoma identification.

**Conclusion:**

Renal sarcomas are rare tumors commonly manifesting as large masses in young patients. A random forest model using demographics and imaging features shows good diagnostic accuracy for discrimination of renal sarcomas from non-sarcoma renal tumors, which might aid in clinical decision-making.

**Key Points:**

• *Renal sarcomas commonly manifest in younger patients as large, complex renal masses.*

• *Compared to non-sarcoma renal tumors, renal sarcomas more frequently demonstrated invasion of the renal vein or inferior vena cava, tumor necrosis, direct invasion of adjacent organs, and contact to renal artery or vein.*

• *Using demographics and standardized imaging features, a random forest showed excellent diagnostic performance for discrimination of sarcoma vs. non-sarcoma renal tumors (AUC = 93.8%, sensitivity = 90.4%, specificity = 76.5%, and PPV = 93.9%).*

**Supplementary Information:**

The online version contains supplementary material available at 10.1007/s00330-021-08201-4.

## Introduction

Renal cancer accounted for 2.2% of all malignant diseases worldwide in 2018 [1]. The most common renal cancer subtypes, such as clear cell or papillary renal cell cancer, originate from renal epithelial cells [2]. One tumoral mechanism linked to an increased likelihood of local invasion and distant metastases is the so-called epithelial-mesenchymal transition (EMT): this process includes disruption of the renal epithelial polarity and barrier integrity, resulting in a mesenchymal phenotype termed sarcomatoid renal cancer [3].

In contrast, sarcomas are rare tumors of mesenchymal origin that can manifest throughout the body [4]. In two older studies, renal sarcomas are exceptionally rare with a prevalence of less than 1% of all renal malignancies [5, 6]. Although there are no recent epidemiological studies on renal sarcomas, unpublished data from the United States National Cancer Database and Surveillance, Epidemiology, and End Results (SEER) database conform the rarity of renal sarcomas contributing < 1% of renal malignancies (unpublished data, 2021).

The prognosis of patients presenting with renal sarcomas is worse compared to non-sarcoma renal tumors, with 5-year overall survival rates of 14.5% reported in one study by Wang and colleagues [6]. This dismal prognosis highlights the clinical need for a timely and accurate diagnosis of renal sarcomas to provide individualized treatment strategies for affected patients.

For renal mass assessment in general, cross-sectional imaging studies, such as computed tomography (CT) and magnetic resonance imaging (MRI), are the cornerstone for initial diagnosis and treatment planning. For renal sarcoma in particular, CT and MRI imaging features have been evaluated in three review articles and numerous case reports [7–9]. Several imaging features unique to renal sarcomas have been described in these studies, such as capsular growth and central calcifications [7–9]. However, the literature does not assess the frequency of these specific features and lacks standardized imaging assessment.

Diagnosing renal sarcomas based on cross-sectional imaging alone remains a radiological challenge given the wide imaging spectrum and overall rarity of the disease, as well as oftentimes non-standardized imaging techniques [7–9]. Still, imaging-based diagnosis of renal sarcoma might optimize clinical management by identifying patients with dismal prognosis and high probability of metastatic disease. Further, international guidelines advocate for risk-adapted perioperative treatment of retroperitoneal sarcomas, including neoadjuvant chemotherapy and radiation, which could play a role in renal sarcoma treatment as well [10]. On the other hand, the role and diagnostic accuracy of renal biopsy in cases with large necrotic tumors, such as renal sarcomas, remains unclear [11].

Therefore, this multicenter study aims to systematically assess imaging features of renal sarcomas, and to present a method for accurate discrimination of renal sarcomas from non-sarcoma renal tumors.

## Material and methods

This retrospective study was approved by the ethics committee of Magdeburg University (Nr 148/20). Imaging assessment and statistical analyses were conducted between September and December 2020.

### Patient cohort

Adult patients diagnosed with sarcomas of renal origin between 1995 and 2018 were included from 11 centers across Europe (Germany, Belgium, Turkey), which identified suitable cases thorough internal database queries. Renal sarcoma diagnosis was based on pathological assessment. Renal sarcoma subtypes were stratified according to the most recent WHO classification of soft-tissue sarcomas [12]. Patients were excluded if they presented with recurrent sarcomas after initial therapy, with renal metastases on non-renal sarcomas, with sarcomas of non-renal origin (i.e., retroperitoneal sarcomas), and if they were younger than 18 years of age.

For the control group, a random sample was drawn from consecutive patients with histopathological diagnosis of non-sarcoma renal tumors receiving imaging of renal tumors at more than 10 German imaging centers between 2016 and 2020. This control group was 4:1 sized in comparison to the renal sarcoma group based on sample size calculations stating that at least *n* = 130 patients were needed to detected medium-sized differences in imaging features across patient subgroups with a power of 80% at an alpha-level of 0.05. Inclusion criteria for the control group were pathological assessment of solid renal tumors in adult patients. Recurrent renal tumors after initial therapy were excluded.

### Image assessment

Image assessment was performed on axial, sagittal, and coronal image slides by one reader with 4 years of experience in abdominal imaging who was blinded to the histopathological diagnosis and case selection process (i.e., number of renal sarcomas and non-sarcoma renal tumors). In unequivocal cases, consensus was reached with a second reader with 15 years of experience in abdominal imaging. Renal tumors were preferably assessed on CT studies, and correlated with MRI studies for evaluation of tumor necrosis and tumor cysts.

Renal tumor appearance and extent were quantified by 21 different features using a checklist, which is provided in Supplemental Table 1. Features were identified from earlier studies on renal tumor imaging in general, and renal sarcoma appearance in specific [7–9, 13].

The RENAL nephrectomy score was used for assessment of renal tumor complexity [13]. The RENAL score was calculated as detailed in Supplemental Table 2 and ranged between 4 and 12 points. Tumor complexity was stratified as “low complexity” (RENAL score 4–6), “intermediate complexity” (RENAL score 7–9), and “high complexity” (RENAL score 10–12). Renal tumors with complete replacement of the renal parenchyma were conservatively rated at RENAL score 12.

### Statistical analyses

Continuous variables were provided as median and inter-quartile-range (IQR), and categorical variables as absolute number and percentage. Across subgroups, continuous variables were compared using the Wilcoxon rank sum test, and categorical variables using the chi-square test.

A random forest machine learning algorithm was used to predict the renal tumor histology as a binary discrimination of “sarcoma vs. non-sarcoma histology” based on imaging and clinical variables as detailed in the Supplemental Table 3. The algorithm was trained using a 10-fold cross-validation (CV). Within this external CV, continuous variables were centered and scaled, and the random forest algorithm was optimized using a 2 × 5-fold internal CV. The random forest tuning parameter “mtry” (number of variables provided at each node) was identified using a grid search. The final random forest model was based on the majority vote over 500 bootstrap samples.

The diagnostic performance was based on out-of-bag samples and quantified using the area-under-the receiver-operating-characteristics curve (AUC; ROC). To provide an AUC-estimate over the 10-fold CV, the median AUC was provided. The Gini index was used to assess variable importance in the random forest models. The Youden Index was utilized to calculate sensitivity, specificity, and the positive predictive value (PPV) from the ROC curve.

Sensitivity analyses were conducted including only imaging features and excluding patient age and gender.

All statistical analyses were performed using R version 3.6.0 and RStudio version 1.3.959 implementing the “caret” package. An alpha-level of 0.05 was considered to indicate statistical significance. All provided *p* values are two-sided.

## Results

### Patient cohort

A total of *n* = 34 renal sarcomas were included and compared to *n* = 136 non-sarcoma renal tumors. Renal sarcoma cases were submitted from *n* = 11 centers across Europe.

Renal sarcoma patients were of younger age compared to non-sarcoma cases (median 55 years, IQR: 45.5–66.8 years versus 67; IQR: 56.8–74 years, *p* < 0.01). In contrast, both histological groups demonstrated a male predominance: renal sarcoma patients were of male gender in 70.6% of the cases versus 66.2% in non-sarcoma renal tumors (*p* = 0.78).

As detailed in Table 1, the most common histological subtypes were leiomyosarcoma (LMS; *n* = 8, 23.5%) and Ewing sarcoma (*n* = 5, 14.7%) in the sarcoma group. In the non-sarcoma group, clear cell renal cell carcinoma (ccRCC; *n* = 81, 59.6%) was the predominant histology, followed by papillary RCC (*n* = 21, 15.4%).

**Table 1:** Histological renal tumor subtypes of included patients

Renal sarcomas (total *n* = 34)			Non-sarcoma renal tumors (total *n* = 136)		
Histological subtype	*n*	Proportion	Histological Subtype	*n*	Proportion
LMS	8	23.5%	ccRCC	81	59.6%
Ewing sarcoma	5	14.7%	Papillary	21	15.4%
Liposarcoma	4	11.8%	Chromophobe	10	7.4%
Dedifferentiated sarcoma	3	8.8%	Oncocytoma	9	6.6%
MFH	3	8.8%	AML	8	5.9%
PNET	3	8.8%	Sarcomatoid RCC	7	5.1%
Synovial sarcoma	3	8.8%			
Angiosarcoma	1	2.9%			
Large cell sarcoma	1	2.9%			
Mesenchymal chondrosarcoma	1	2.9%			
Osteosarcoma	1	2.9%			
Spindle cell sarcoma	1	2.9%			

*LMS* leiomyosarcoma, *MFH* malignant fibrous histiocytoma, *PNET* primitive neuroectodermal tumor, *RCC* renal cell carcinoma, *ccRCC* clear cell renal cell carcinoma, *AML* angiomyolipoma

### CT and MR imaging

Of 34 included renal sarcoma patients, a total of 26 (76%) were exclusively imaged using CT studies, and 6 patients (18%) received MR imaging only. Combined CT and MRI studies were available in 2 cases. All imaging studies were performed by using the administration of intravenous iodinated or gadolinium-based contrast media. Among the CT studies, a minority were performed using a triphasic imaging protocol including native, corticomedullary, and nephrogenic phase (24.2%). Another 21.2% of CT studies were performed in corticomedullary and nephrogenic phase.

Among the non-sarcoma renal tumors, all CT studies were performed after intravenous administration of iodinated contrast media. A triphasic CT protocol was performed in *n* = 56 patients (41.2%), while another *n* = 53 CT studies (39%) were conducted in corticomedullary and nephrogenic phase. A total of 27 CT studies (19.9%) were performed only in corticomedullary or only nephrogenic phase.

### RENAL score

A higher median RENAL score was observed in renal sarcomas (10 points) compared to non-sarcoma renal tumors (9 points, *p* < 0.01). Accordingly, renal tumor complexity was rated as “high” in 79.4% of the renal sarcomas compared to 25.7% non-sarcoma renal tumors (*p* < 0.01). Additional details on the RENAL score and its components are provided in Supplemental Table 4.

### Renal sarcoma imaging features

As summarized in Table 2, renal sarcomas predominantly presented as right sided renal masses with larger diameter compared to non-sarcoma renal tumors and were more likely to have an irregular tumor shape as well as ill-defined tumor margins (*p* < 0.001, each). Renal sarcomas more frequently showed direct contact to renal artery or veins, as well as invasion of the renal vein or inferior vena cava, tumor necrosis, and direct invasion of adjacent organs (*p* < 0.05, each). Imaging features of the 3 most common renal sarcoma subtypes are provided in Supplemental Table 5.

**Table 2:** Comparison of imaging features of renal sarcomas and non-sarcoma renal tumors

Parameter	Level	Renal sarcoma	Non-sarcoma renal tumors	*p* value
*n*		34	136	
Laterality				< 0.01
	Right	21 (61.8%)	48 (35.3%)	
	Left	12 (35.3%)	88 (64.7%)	
	Bilateral	1 (2.9%)	0 (0.0%)	
Maximum diameter [mm]				< 0.01
	Median (IQR)	108 (83.5–163)	43 (30–62.2)	
Complete renal replacement by tumor				0.19
	No	32 (94.1%)	135 (99.3%)	
	Yes	2 (5.9%)	1 (0.7%)	
Tumor shape				< 0.01
	Irregular	21 (61.8%)	45 (33.1%)	
	Oval	8 (23.5%)	33 (24.3%)	
	Round	5 (14.7%)	58 (42.6%)	
Tumor margins				< 0.01
	Ill-defined	23 (67.6%)	47 (34.6%)	
	Well-defined	11 (32.4%)	89 (65.4%)	
Tumor contact to renal artery or vein				< 0.01
	No	6 (17.6%)	63 (46.3%)	
	Yes	28 (82.4%)	73 (53.7%)	
Renal vein invasion				< 0.01
	No	15 (44.1%)	114 (83.8%)	
	Yes	19 (55.9%)	22 (16.2%)	
IVC invasion				< 0.01
	No	24 (70.6%)	132 (97.1%)	
	Yes	10 (29.4%)	4 (2.9%)	
Tumor necrosis				< 0.01
	None	9 (26.5%)	76 (55.9%)	
	Sporadic (< 50%)	12 (35.3%)	39 (28.7%)	
	Extensive (≥ 50%)	13 (38.2%)	21 (15.4%)	
Calcification				0.63
	None	30 (88.2%)	126 (92.6%)	
	Sporadic (< 50%)	4 (11.8%)	10 (7.4%)	
Macroscopic tumor fat				0.44
	None	31 (91.2%)	131 (96.3%)	
	Sporadic (< 50%)	2 (5.9%)	3 (2.2%)	
	Extensive (≥ 50%)	1 (2.9%)	2 (1.5%)	
Perinephric hemorrhage				0.45
	No	33 (97.1%)	136 (100.0%)	
	Yes	1 (2.9%)	0 (0.0%)	
Hydronephrosis				0.66
	No	26 (76.5%)	111 (81.6%)	
	Yes	8 (23.5%)	25 (18.4%)	
Continuous organ invasion				< 0.01
	None	21 (61.8%)	132 (97.1%)	
	Adrenal	3 (8.8%)	3 (2.2%)	
	Liver	3 (8.8%)	1 (0.7%)	
	Spleen	3 (8.8%)	0 (0.0%)	
	Other	4 (11.8%)	0 (0.0%)	

*IVC* inferior vena cava

**Fig. 1:**
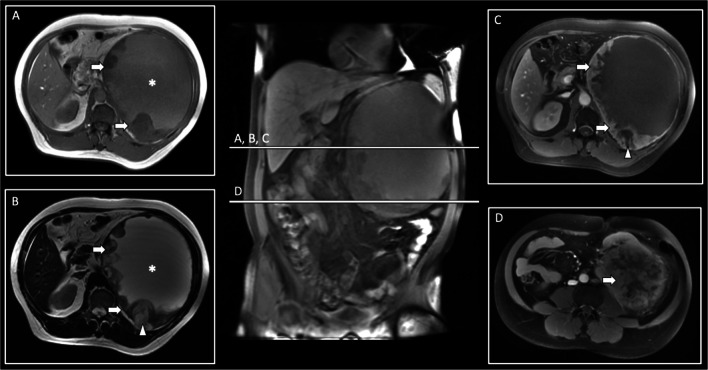
MRI case study of a 49-year-old male patient presenting with a left-sided cystic renal mass of 204-mm diameter with inferior renal displacement. T1w imaging (A) demonstrates intermediate central signal intensity (SI; star) and low SI nodular rim (arrows). T2w imaging (B) confirms a high-SI central cystic (star) as well as necrotic components (arrowhead) within the nodular rim (arrows). Post-contrast T1w fat-saturated imaging (C, D) confirms an enhancing nodular rim (arrows) with necrotic components (arrowhead), and mass-like appearance of the left renal upper pole (arrow, D). Histopathological analyses revealed a renal osteosarcoma with central cystic with myxoid content, wall-associated necrotic areas, and osseous as well as cartilaginous components

**Fig. 2:**
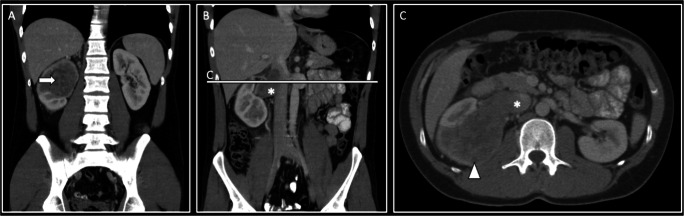
Venous-phase contrast-enhanced CT case study of a 61-year-old male patient presenting with a right-sided solid renal mass of 75mm diameter. Coronal images (A) demonstrate a primarily endophytic, heterogeneous mass (arrow). Invasion of the renal vein and inferior vena cava is evident on coronal (B; star) and axial images (C; star). Sporadic hypodense areas are noted on axial images (C; arrowhead), in keeping with tumor necrosis. Histopathological analyses revealed a renal Ewing sarcoma with rosette-like tumoral proliferations and necrotic areas

Representative case studies demonstrating renal sarcoma imaging features are provided in Figs. 1 and 2.

### Prediction of renal sarcoma histology

**Fig. 3:**
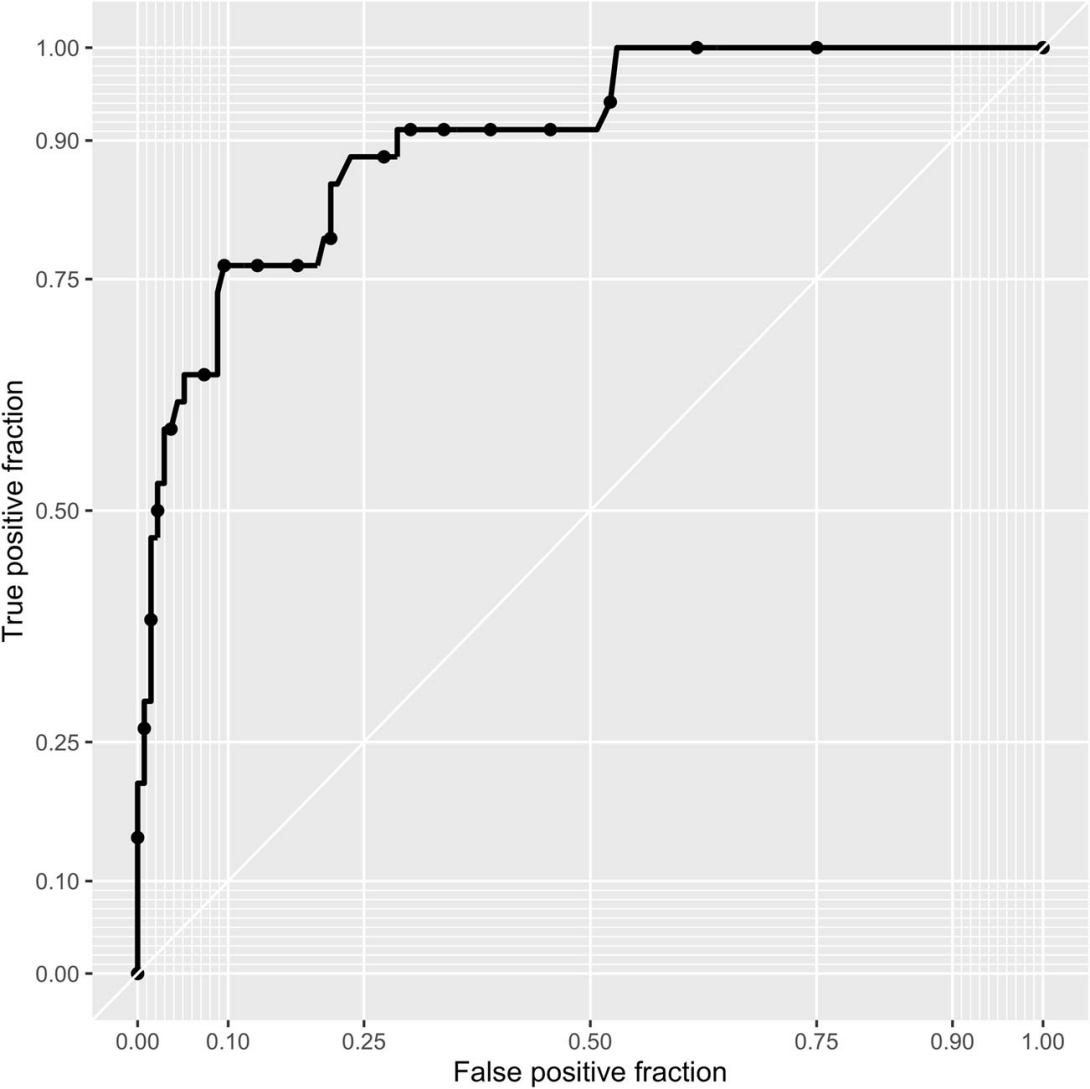
Receiver-operating-characteristics curve obtained from out-of-bag sample predictions of the random forest algorithm across 10-fold cross-validation

Applying a random forest algorithm for discrimination of sarcoma vs. non-sarcoma renal tumors yielded a median AUC of 93.8% in the out-of-bag samples. Using the Youden index to dichotomize predictions, a sensitivity, specificity, and PPV of 90.4%, 76.5%, and 93.9% were obtained, respectively. The corresponding ROC curve is presented in Fig. 3.

**Fig. 4:**
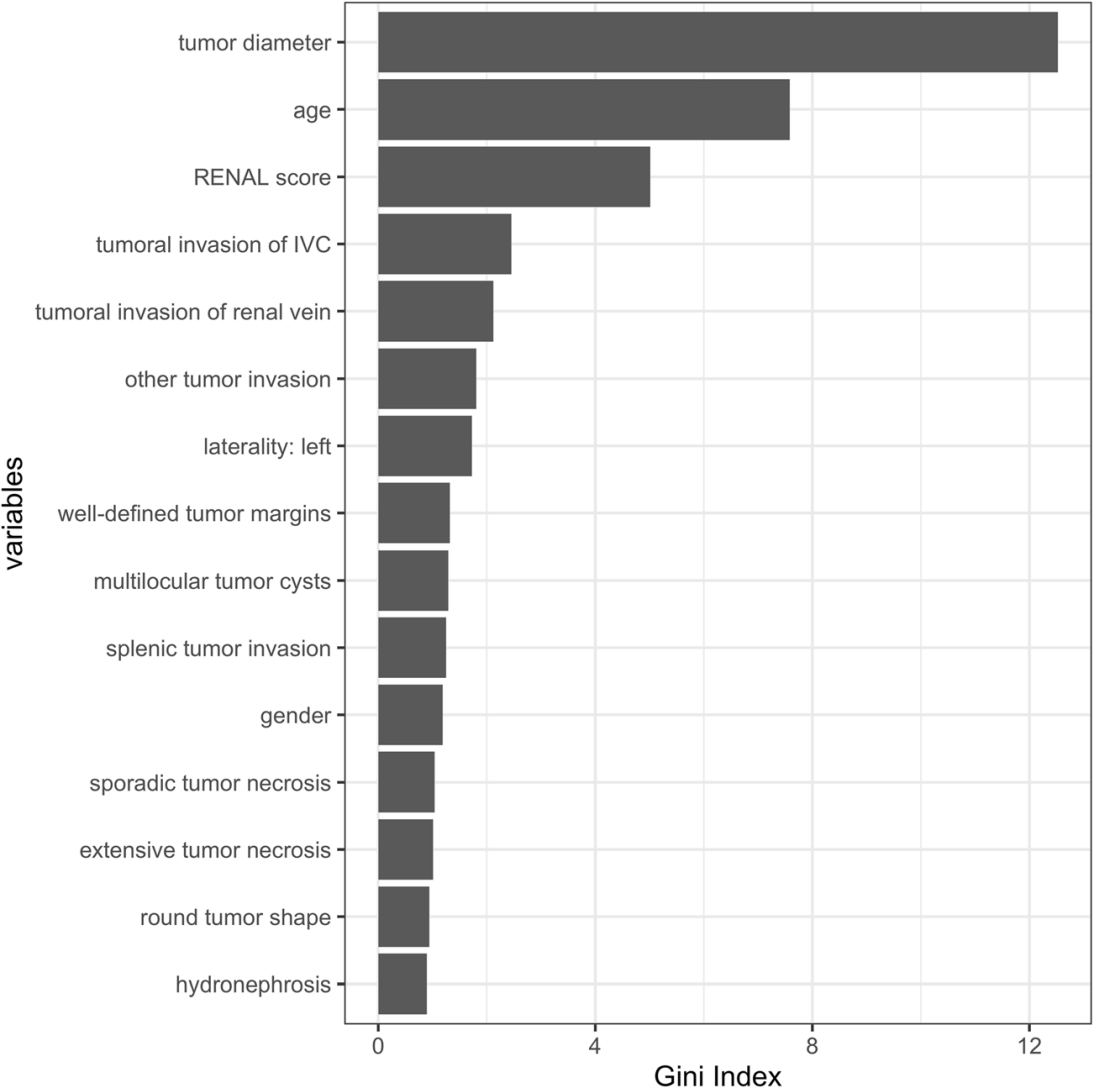
Variable importance measured using the random forest Gini Index

Utilizing the Gini Index, the highest variable importance was detected for tumor diameter and patient age. Figure 4 summarizes the most 15 most important variables in the random forest model.

In a separate sensitivity including only imaging features and excluding patient age and gender, the random forest model yielded an AUC = 91.9%, reaching a sensitivity, specificity, and PPV of 80.1%, 85.3%, and 95.6%, respectively.

## Discussion

Renal sarcomas are rare mesenchymal tumors with a reported incidence < 1% of all renal malignancies [5, 6]. Although renal sarcomas are associated with a dismal prognosis, so far there were no efforts to systematically describe their imaging features and to clinically discriminate renal sarcomas from non-sarcoma renal tumors.

Including data from 11 European centers, this study reports on a total of 34 patients with renal sarcomas, with leiomyosarcoma and Ewing sarcoma being the most common histological subtypes (23.5% and 14.7%, respectively). Given the multicenter design and long accrual period, the resulting database was heterogeneous regarding imaging techniques.

Compared to non-sarcoma renal tumors, renal sarcomas generally manifested as more complex renal masses in younger patients and were more complex renal masses with larger diameter and irregular shape as well as ill-defined margins, compared to non-sarcoma renal tumors. Renal sarcomas also demonstrated a more aggressive local growth pattern with more frequent invasion of the renal vein or inferior vena cava, tumor necrosis, direct invasion of adjacent organs, and contact to renal artery or vein.

Using imaging features and demographics, a random forest algorithm showed good discrimination of renal sarcomas versus non-sarcoma renal tumors with an AUC = 93.8%. Using a robust 10-fold cross-validation approach, the diagnostic performance was based on out-of-bag CT studies that were not available to the random forest model during training phase, which could therefore be considered independent CT samples during the respective cross-validation iteration. The random forest identified tumor diameter, patient age, and RENAL score as the most important imaging features for renal sarcoma identification, highlighting the clinical applicability of the proposed algorithm. In separate analysis using imaging features only, the statistical model still yielded a high diagnostic performance with AUC = 91.9%, thus corroborating the diagnostic importance of imaging features for renal sarcoma identification.

The patient cohorts assessed in this study compares well to the published literature. For example, Wang et al reported a median patient age of 42 years and a predominant leiomyosarcoma histology (39%) in the majority of renal sarcoma cases [6].

Further, the random sample of non-sarcoma renal tumors in our study yielded a distribution of histological subtypes that is in line with earlier studies, reporting clear cell RCC as the most frequent subtype in 75–80% of malignant cases, followed by papillary RCC in 10–15% [2]. In this context, it has to be highlighted that benign AMLs and oncocytomas were purposely included in our study to better reflect diagnostic challenges in assessment of renal masses of uncertain differentiation, such as discrimination of fat-rich AMLs from fat-containing renal liposarcomas. Similarly, sarcomatoid RCCs were purposely sampled to compare imaging features of RCCs which underwent epithelial-mesenchymal transition to those of primary mesenchymal renal sarcomas. Further, the broad inclusion of various non-sarcoma histological subtypes maximized the generalizability and clinical applicability of the random forest algorithm to discriminate between renal sarcomas and non-sarcoma renal tumors.

Renal sarcoma imaging features have so far been described in several case reports and 3 larger reviews [7–9]. The literature suggests that the imaging features of renal sarcomas vary with the numerous renal sarcoma subtypes. Comparing the most common renal sarcoma subtypes in our study, the only specific imaging features was a higher proportion of macroscopic fat for liposarcomas (75%) versus Ewing sarcoma and leiomyosarcoma (each 0%, *p* = 0.02). Still, missing statistical significance in imaging feature subgroup analyses might be attributable to the small subgroup sizes resulting from the overall rarity of renal sarcomas.

In general, the literature supports our finding that renal sarcomas present with a larger tumor diameter compared to non-sarcoma renal tumors. For example, Lalwani et al noted an average renal sarcoma size between 55 and 230 mm at time of diagnosis, while Karaosmanoglu et al specified a mean size of 80 mm for renal synovial sarcoma [8, 9]. The locally aggressive growth of renal sarcomas observed in our study is in line with the literature, i.e., reporting inferior vena cava tumor thrombus in patients with renal liposarcomas, fibrosarcomas, leiomyosarcomas, malignant fibrous histiocytoma, and synovial sarcomas [7, 14, 15]. Renal sarcoma patients frequently presented with tumor necrosis in our cohort (73.5%), which has been described to be a common feature of renal sarcomas [16–18]. Still, the high proportion of continuous renal sarcoma invasion of adjacent organs, such as spleen or liver, detected in 38.2% of patients in this study, has not been reported so far.

Among 34 included renal sarcoma cases, only one case showed imaging features that were previously described as specific for renal sarcomas, demonstrating a capsular growth pattern in a patient with renal liposarcoma [14]. In contrast, imaging features such as tumoral calcification and fat that some authors reported to be imaging hallmarks of renal sarcomas were observed in both renal sarcoma and non-sarcoma renal tumors in this study, thus limiting their discriminatory value [7–9].

With an AUC = 93.8%, the random forest trained in this study demonstrated a good diagnostic performance, highlighting the potential clinical benefit of this method for accurate renal sarcoma classification. Especially given the low incidence of renal sarcomas and the resulting unfamiliarity of radiologists with associated imaging features, radiological identification of renal sarcomas might be improved by utilization of the proposed random forest algorithm.

In light of the dismal prognosis of renal sarcomas and their propensity to metastasize, an accurate and timely image-based diagnosis might improve patient management. Specifically, the ESMO guideline recommends a risk-adapted perioperative treatment of patients with retroperitoneal sarcoma, including neoadjuvant chemotherapy and radiation, which might play a role in renal sarcomas as well [10]. On the other hand, the role and diagnostic accuracy of renal biopsy in cases with large necrotic tumors, such as renal sarcomas, remains unclear [11]. There are several limitations to this study. First, given the overall rarity of renal sarcomas, the sample size of specific histological subgroups was small, with resulting comparably low statistical power to detect differences in imaging feature subgroup analyses. Second, not all patients received imaging studies according to standardized protocols or three-phasic studies, as recommended for CT assessment of renal masses. This might reduce their interpretability regarding imaging features such as vascularization or tumor necrosis. Third, no external cohort was available to validate the proposed random forest algorithm, which might have resulted in statistical overfitting. Still, a conservative 10-fold external cross-validation was used to minimize potential overfitting, and the accrual of an adequately sized external cohort seems impractical given the rarity of renal sarcomas.

Further, the varying accrual periods of the renal sarcoma and non-sarcoma subgroups might have introduced bias through varying standards in image acquisition, although imaging features analyzed in this study were assessable in all patients from both cohorts.

Finally, there is a lack of studies on renal sarcomas from the last 5 years, and thus, newest epidemiological developments as well as advancements in renal tumor diagnosis and treatment might not be reflected by the cited references.

## Conclusions

Renal sarcomas are rare mesenchymal renal tumors that tend to manifest as large, complex renal masses in young patients. A random forest algorithm trained on standardized imaging features and patient demographics shows good diagnostic accuracy for discrimination of renal sarcomas from non-sarcoma renal tumors using a cross-validation approach, though limited by the low number of patients. Tumor diameter and RENAL score were the most relevant imaging features to identify renal sarcomas.

This algorithm might aid in timely and accurate diagnosis of renal sarcomas in clinical practice in an effort to optimize and individualize treatment of patients with renal cancer.

## Supplementary Information

ESM 1 (DOCX 34 kb)

## Abbreviations

AML: Angiomyolipoma
AUC: Area under the ROC curve
ccRCC: Clear cell renal cell carcinoma
CT: Computed tomography
IQR: Interquartile range
MRI: Magnetic resonance imaging
RCC: Renal cell carcinoma
ROC: Receiver operating characteristics curve

## Declarations

### Guarantor

The scientific guarantor of this publication is Alexey Surov.

### Statistics and biometry

Two of the authors have significant statistical expertise.

### Methodology

• observational
