# Supplementary material for: Primary renal sarcomas: imaging features and discrimination from non-sarcoma renal tumors
Source: Eur Radiol. 2021 Jul 31;32(2):981–9. doi: 10.1007/s00330-021-08201-4 (PMC8794936; doi:10.1007/s00330-021-08201-4)
Supplement: Supplementary file 1 — (DOCX 34 kb) [file 330_2021_8201_MOESM1_ESM.docx]

**Supplemental material**

Supplemental Table 1: Features for quantification of renal tumor appearance and extent.

| **parameter** | **levels** | **details** |
| --- | --- | --- |
| laterality | - right - left - bilateral |  |
| largest diameter | - <=4cm - 4-7cm - >=7cm | largest diameter in any single plane; RENAL score component |
| exophytic versus endophytic component | - >50% exophytic - <50% exophytic - entirely endophytic | percentage of renal tumor compared to renal parenchyma;  RENAL score component |
| Nearness to collecting system or sinus | - <=4mm - 4-7mm - >=7mm | measured as shortest distance between renal tumor and collecting system/renal sinus;  RENAL score component |
| anterior or posterior location | - anterior - posterior - neither | location of renal tumor relative to coronal plane at level of hilar vessels; RENAL score component |
| polar location | - Entirely above or below polar lines - Crosses a polar line - >50% across polar line - Crosses axial renal midline - Entirely between polar lines | RENAL score component |
| tumor contact to renal artery or vein | - no - yes | RENAL score component |
| renal vein invasion | - no - yes | assesses tumoral invasion of the renal vein |
| IVC invasion | - no - yes | assesses tumoral invasion of the renal vein |
| maximum diameter | - continuous [mm] | maximum renal tumor diameter in any single plane |
| complete renal replacement by tumor | - no - yes | assesses whether the renal tumor completely replaces the renal parenchyma |
| macroscopic tumor fat | - none - sporadic (<50%) - extensive (>50%) | assessed by enhancement of less than -10 Hounsfield units; percentages refer to renal tumor volume comprised of fat |
| tumor calcifications | - none - sporadic (<50%) - extensive (>50%) | percentages refer to renal tumor volume comprised of fat |
| tumor cysts | - none - solitary - multilocular |  |
| tumor necrosis | - none - sporadic (<50%) - extensive (>50%) | defined as absence of enhancement with central location on CT; or absence of enhancement with high T2w signal, low T1w signal and diffusion restriction (if available) on MRI |
| perinephric hemorrhage | - no - yes |  |
| tumor shape | - round - oval - irregular |  |
| tumor margins | - well-defined - ill-defined |  |
| hydronephrosis | - no - yes |  |
| tumor enhancement pattern | - homogeneous - heterogeneous |  |
| continuous organ invasion | - none - adrenal - liver - spleen - other |  |

Supplemental Table 2: RENAL score components and calculation (adapted from Kutikov et al. 2009)

| **RENAL score component** | **+1 point** | **+2 points** | **+3 points** |
| --- | --- | --- | --- |
| **Radius** | <=4cm | 4-7cm | >=7cm |
| **Exophytic/Endophytic** | >50% exophytic | <50% exophytic | entirely endophytic |
| **Nearness to renal sinus or collecting system** | >=7mm | 4-7mm | <=4mm |
| **Polar location** | Entirely above or below polar lines | Crosses a polar line | >50% across polar line; entirely between polar lines; or crosses axial renal midline |

Supplemental table 3: Imaging and clinical variables used for training and testing of the random forest algorithm for prediction of renal tumor histology (sarcoma vs. non-sarcoma). RENAL score components were excluded due to missing values in cases with complete replacement of the renal parenchyma.

| **variables** |
| --- |
| age |
| gender |
| laterality |
| largest diameter |
| RENAL score |
| tumor contact to renal artery or vein |
| renal vein invasion |
| IVC invasion |
| maximum diameter |
| complete renal replacement by tumor |
| macroscopic tumor fat |
| tumor calcifications |
| tumor cysts |
| tumor necrosis |
| perinephric hemorrhage |
| tumor shape |
| tumor margins |
| hydronephrosis |
| tumor enhancement pattern |
| continuous organ invasion |

Supplemental table 4: RENAL score and RENAL score components. Missing values are due to complete tumor replacement of renal parenchyma in 3 patients.

| parameter | level | total | renal sarcoma | non-sarcoma renal tumors | p value |
| --- | --- | --- | --- | --- | --- |
| n |  | 170 | 34 | 136 |  |
| RENAL score |  |  |  |  | < 0.01 |
|  | mean ± sd | 8.62 ± 1.81 | 9.79 ± 1.67 | 8.33 ± 1.73 |  |
|  | median (IQR) | 9 (8-10) | 10 (10-11) | 9 (7-10) |  |
| renal tumor complexity |  |  |  |  | < 0.01 |
|  | low | 23 (13.5%) | 2 (5.9%) | 21 (15.4%) |  |
|  | intermediate | 85 (50.0%) | 5 (14.7%) | 80 (58.8%) |  |
|  | high | 62 (36.5%) | 27 (79.4%) | 35 (25.7%) |  |
| largest diameter |  |  |  |  | < 0.01 |
|  | <=4cm | 64 (37.6%) | 2 (5.9%) | 62 (45.6%) |  |
|  | 4-7cm | 52 (30.6%) | 5 (14.7%) | 47 (34.6%) |  |
|  | >=7cm | 54 (31.8%) | 27 (79.4%) | 27 (19.9%) |  |
| exophytic versus endophytic component |  |  |  |  | 0.04 |
|  | >50% exophytic | 89 (53.3%) | 23 (71.9%) | 66 (48.9%) |  |
|  | <50% exophytic | 69 (41.3%) | 9 (28.1%) | 60 (44.4%) |  |
|  | entirely endophytic | 9 (5.4%) | 0 (0.0%) | 9 (6.7%) |  |
|  | missing | 3 | 2 | 1 |  |
| nearness to collecting system or renal sinus |  |  |  |  | 0.37 |
|  | >=7mm | 16 (9.6%) | 3 (9.4%) | 13 (9.6%) |  |
|  | 4-7mm | 8 (4.8%) | 0 (0.0%) | 8 (5.9%) |  |
|  | <=4mm | 143 (85.6%) | 29 (90.6%) | 114 (84.4%) |  |
|  | missing | 3 | 2 | 1 |  |
| anterior or posterior location |  |  |  |  | 0.04 |
|  | anterior | 44 (26.3%) | 4 (12.5%) | 40 (29.6%) |  |
|  | posterior | 31 (18.6%) | 4 (12.5%) | 27 (20.0%) |  |
|  | neither | 92 (55.1%) | 24 (75.0%) | 68 (50.4%) |  |
|  | missing | 3 | 2 | 1 |  |
| polar location |  |  |  |  | < 0.01 |
|  | >50% across polar line | 7 (4.2%) | 2 (6.2%) | 5 (3.7%) |  |
|  | Crosses a polar line | 45 (26.9%) | 1 (3.1%) | 44 (32.6%) |  |
|  | Crosses axial renal midline | 84 (50.3%) | 27 (84.4%) | 57 (42.2%) |  |
|  | Entirely above or below | 31 (18.6%) | 2 (6.2%) | 29 (21.5%) |  |
|  | missing | 3 | 2 | 1 |  |

Supplemental table 5: imaging features of the three most frequent renal sarcoma subtypes.

| parameter | level | total | Ewing sarcoma | liposarcoma | LMS | p value |
| --- | --- | --- | --- | --- | --- | --- |
| n |  | 17 | 5 | 4 | 8 |  |
| RENAL score |  |  |  |  |  | 0.09 |
|  | median (IQR) | 10 (10-11) | 11 (10-11) | 10 (9.5-10) | 10 (10-11) |  |
| laterality |  |  |  |  |  | 0.83 |
|  | right | 12 (70.6%) | 3 (60.0%) | 3 (75.0%) | 6 (75.0%) |  |
|  | left | 5 (29.4%) | 2 (40.0%) | 1 (25.0%) | 2 (25.0%) |  |
|  | bilateral | 0 (0.0%) | 0 (0.0%) | 0 (0.0%) | 0 (0.0%) |  |
| maximum diameter [mm] |  |  |  |  |  | 0.38 |
|  | median (IQR) | 106 (87-149) | 109 (87-220) | 115 (89.2-165) | 102 (77.8-120) |  |
| complete renal replacement by tumor |  |  |  |  |  | 0.28 |
|  | no | 16 (94.1%) | 4 (80.0%) | 4 (100.0%) | 8 (100.0%) |  |
|  | yes | 1 (5.9%) | 1 (20.0%) | 0 (0.0%) | 0 (0.0%) |  |
| tumor shape |  |  |  |  |  | 0.46 |
|  | irregular | 14 (82.4%) | 3 (60.0%) | 4 (100.0%) | 7 (87.5%) |  |
|  | oval | 2 (11.8%) | 1 (20.0%) | 0 (0.0%) | 1 (12.5%) |  |
|  | round | 1 (5.9%) | 1 (20.0%) | 0 (0.0%) | 0 (0.0%) |  |
| tumor margins |  |  |  |  |  | 0.65 |
|  | ill-defined | 15 (88.2%) | 4 (80.0%) | 4 (100.0%) | 7 (87.5%) |  |
|  | well-defined | 2 (11.8%) | 1 (20.0%) | 0 (0.0%) | 1 (12.5%) |  |
| tumor contact to renal artery or vein |  |  |  |  |  | 0.51 |
|  | no | 2 (11.8%) | 0 (0.0%) | 1 (25.0%) | 1 (12.5%) |  |
|  | yes | 15 (88.2%) | 5 (100.0%) | 3 (75.0%) | 7 (87.5%) |  |
| renal vein invasion |  |  |  |  |  | 0.58 |
|  | no | 5 (29.4%) | 1 (20.0%) | 2 (50.0%) | 2 (25.0%) |  |
|  | yes | 12 (70.6%) | 4 (80.0%) | 2 (50.0%) | 6 (75.0%) |  |
| IVC invasion |  |  |  |  |  | 0.22 |
|  | no | 11 (64.7%) | 3 (60.0%) | 4 (100.0%) | 4 (50.0%) |  |
|  | yes | 6 (35.3%) | 2 (40.0%) | 0 (0.0%) | 4 (50.0%) |  |
| tumor necrosis |  |  |  |  |  | 0.26 |
|  | none | 4 (23.5%) | 0 (0.0%) | 2 (50.0%) | 2 (25.0%) |  |
|  | sporadic (<50%) | 6 (35.3%) | 2 (40.0%) | 0 (0.0%) | 4 (50.0%) |  |
|  | extensive (>=50%) | 7 (41.2%) | 3 (60.0%) | 2 (50.0%) | 2 (25.0%) |  |
| calcification |  |  |  |  |  | 0.47 |
|  | none | 17 (100.0%) | 5 (100.0%) | 4 (100.0%) | 8 (100.0%) |  |
|  | sporadic (<50%) | 0 (0.0%) | 0 (0.0%) | 0 (0.0%) | 0 (0.0%) |  |
| macroscopic tumor fat |  |  |  |  |  | *0.02* |
|  | none | 14 (82.4%) | 5 (100.0%) | 1 (25.0%) | 8 (100.0%) |  |
|  | sporadic (<50%) | 2 (11.8%) | 0 (0.0%) | 2 (50.0%) | 0 (0.0%) |  |
|  | extensive (>=50%) | 1 (5.9%) | 0 (0.0%) | 1 (25.0%) | 0 (0.0%) |  |
| perinephric hemorrhage |  |  |  |  |  | 0.47 |
|  | no | 17 (100.0%) | 5 (100.0%) | 4 (100.0%) | 8 (100.0%) |  |
|  | yes | 0 (0.0%) | 0 (0.0%) | 0 (0.0%) | 0 (0.0%) |  |
| hydronephrosis |  |  |  |  |  | 0.16 |
|  | no | 11 (64.7%) | 4 (80.0%) | 1 (25.0%) | 6 (75.0%) |  |
|  | yes | 6 (35.3%) | 1 (20.0%) | 3 (75.0%) | 2 (25.0%) |  |
| continuous organ invasion |  |  |  |  |  | 0.31 |
|  | none | 10 (58.8%) | 3 (60.0%) | 1 (25.0%) | 6 (75.0%) |  |
|  | adrenal | 2 (11.8%) | 1 (20.0%) | 1 (25.0%) | 0 (0.0%) |  |
|  | liver | 2 (11.8%) | 1 (20.0%) | 0 (0.0%) | 1 (12.5%) |  |
|  | spleen | 0 (0.0%) | 0 (0.0%) | 0 (0.0%) | 0 (0.0%) |  |
|  | other | 3 (17.6%) | 0 (0.0%) | 2 (50.0%) | 1 (12.5%) |  |

LMS: leiomyosarcoma; IVC: inferior vena cava
